# Monitoring the hepatitis C epidemic in England and evaluating intervention scale‐up using routinely collected data

**DOI:** 10.1111/jvh.13063

**Published:** 2019-02-28

**Authors:** Ross J. Harris, Helen E. Harris, Sema Mandal, Mary Ramsay, Peter Vickerman, Matthew Hickman, Daniela De Angelis

**Affiliations:** ^1^ Statistics Modelling and Economics Department National Infection Service Public Health England London UK; ^2^ Immunisation, Hepatitis and Blood Safety Department National Infection Service Public Health England London UK; ^3^ Population Health Sciences Bristol Medical School University of Bristol Bristol UK; ^4^ MRC Biostatistics Unit Cambridge Institute of Public Health Cambridge UK

**Keywords:** back calculation, direct‐acting antiviral treatment, disease burden, people who inject drugs, surveillance data

## Abstract

In England, 160 000 individuals were estimated to be chronically infected with hepatitis C virus (HCV) in 2005 and the burden of severe HCV‐related liver disease has increased steadily for the past 15 years. Direct‐acting antiviral treatments can clear infection in most patients, motivating HCV elimination targets. However, the current burden of HCV is unknown and new methods are required to monitor progress. We employed a Bayesian back‐calculation approach, combining data on severe HCV‐related liver disease and disease progression, to reconstruct historical HCV incidence and estimate current prevalence in England. We explicitly modelled infections occurring in people who inject drugs, the key risk group, allowing information on the size of this population and surveillance data on HCV prevalence to inform recent incidence. We estimated that there were 143 000 chronic infections in 2015 (95% credible interval 123 000‐161 000), with 34% and 54% in those with recent and past injecting drug use, respectively. Following the planned scale‐up of new treatments, chronic infections were predicted to fall to 113 400 (94 900‐132 400) by the end of 2018 and to 89 500 (71 300‐108 600) by the end of 2020. Numbers developing severe HCV‐related liver disease were predicted to fall by at least 24% from 2015 to 2020. Thus, we describe a coherent framework to monitor progress using routinely collected data, which can be extended to incorporate additional data sources. Planned treatment scale‐up is likely to achieve 2020 WHO targets for HCV morbidity, but substantial efforts will be required to ensure that HCV testing and patient engagement are sufficiently high.

## Abstract 2

People who inject drugs are at high risk of acquiring hepatitis C infection, many of whom will develop cirrhosis and severe liver disease. We use surveillance data to model this process, estimating the number of HCV‐infected people in England and predicted impact of new treatments. We estimate 143,000 people had chronic infection in 2015 and predict that this will fall to around 90,000 in 2020.

**Abbreviations**
CrI: credible interval
ESLD: end‐stage liver disease
HCC: hepatocellular carcinoma
HCV: Hepatitis C virus
HES: Hospital Episode Statistics
MPES: multi‐parameter evidence synthesis
NTA: National Treatment Agency
PWID: people who inject drugs
SVR: sustained viral response
UAM: Unlinked Anonymous Monitoring

## 1 INTRODUCTION

Hepatitis C virus (HCV) is a blood‐borne infection that causes progressive fibrosis of the liver and can lead to cirrhosis and hepatocellular carcinoma (HCC). It has a long incubation time, with many individuals remaining asymptomatic for decades, although progression tends to accelerate with age.^1^, ^2^ The majority of new infections in England occur in people who inject drugs (PWID).^3^ The size of this group was estimated to be growing rapidly throughout the 1980s and 1990s,^4^ with over 50% estimated to be HCV antibody positive.^5^ Growing numbers of people with chronic HCV infection are now developing severe liver disease, with the burden predicted to rise over the next decade.^6^ However, new treatments can clear the virus in the majority of those infected, including those with advanced disease.^7^, ^8^ Such is the current optimism that many countries are in the process of setting timelines for eliminating HCV as a major public health threat by 2030 or before,^9^ as set out by the World Health Organization Global Health Sector Strategy for eliminating viral hepatitis.^10^

Timely estimates of chronic prevalence and disease burden are needed for public health planning and monitoring progress towards the eventual elimination of HCV. Opportunistic testing or population surveys are often used to estimate national prevalence.^11^, ^12^, ^13^ Surveys in general are at risk of over‐ or under‐representing PWID (those currently injecting or who have temporarily ceased to inject) and ex‐PWID (those who have injected in the past, but have permanently ceased). For instance, the sampling frame may consist of those registered with the national health system^12^ or who are not homeless or incarcerated.^13^ In Scotland, prevalence estimates were derived on the basis of multi‐parameter evidence synthesis (MPES), which combines data from different sources and estimates prevalence in different risk groups.^14^ The future course of the epidemic and impact of treatment on disease burden has been investigated through natural history models of HCV‐related disease progression. Such models typically make inferences using data on disease endpoints, which are modelled via back‐calculation or used to calibrate mathematical models.^15^, ^16^, ^17^

Two approaches previously used in England are MPES models to estimate the prevalence of HCV^18^, ^19^ and back‐calculation models based on trends in severe HCV‐related liver disease.^6^, ^20^ Key limitations for the MPES approach are that some of the data sources are collected infrequently and information on ex‐PWID is limited.^3^, ^21^ Conversely, the back‐calculation model can be used to reconstruct the historical pattern of HCV infections, on the basis of the time between infection and disease endpoints. However, estimates of recent incidence, and therefore overall prevalence, are more uncertain. We therefore combine features of these two approaches by extending the back‐calculation model to include an additional state representing susceptible PWID, thus explicitly modelling acquisition of infection. The advantage of this approach is that data on the size of the PWID population and on the proportion of HCV‐infected PWID can be incorporated, to better inform recent HCV incidence and estimated prevalence.

## 2 MATERIALS AND METHODS

We combined data on HCV‐related end‐stage liver disease (ESLD) and HCC, disease progression rates, the number of PWID and seroprevalence data on the proportion of HCV‐infected PWID within the extended back‐calculation model. Data on diagnosis and treatment were included in the model to obtain estimates of diagnosed and undiagnosed chronic infections and make predictions of prevalence and disease burden under current and planned treatment levels. The focus here was on infections occurring through injecting drug use, although we also included incidence of infection via other routes in those who have never injected, subdivided into those of South Asian and other ethnicities, the former having a higher risk of HCV infection.^19^

### 2.1 Data sources

The key data for the back‐calculation model were first presentations of ESLD or HCC in patients with HCV, available from Hospital Episode Statistics (HES). Data were available from 2004 to 2016 and were aggregated to 10‐year birth cohorts for analysis. In the base model, we restricted data to the 2011 to 2016 period due to potential reporting biases in earlier periods, which we discuss subsequently. We use information on age‐specific disease progression from the Trent cohort of patients referred for tertiary care in England, which indicate that around 12% (95% CI: 6%‐22%) of individuals will progress to cirrhosis within 20 years of infection.^2^ Progression from compensated cirrhosis to ESLD and HCC is informed by other studies and previous work.^6^, ^22^, ^23^, ^24^

The yearly Unlinked Anonymous Monitoring (UAM) survey provides data on HCV antibody prevalence in PWID.^5^, ^25^ PWID attending low threshold (needle‐exchange) or treatment services are invited to self‐complete a questionnaire on injecting duration and risk behaviour and provide a serological sample, which is tested for HCV antibodies. We used data from 2000 to 2015, aggregated by survey year and injecting duration, the latter corresponding to time at risk. These data inform annual probabilities of chronic infection in PWID via a force of infection model,^26^ on the assumption that 24% of infections spontaneously clear.^27^

Research commissioned by the former National Treatment Agency (NTA) estimated that in the financial year 2010/11, 93 401 people aged 15‐64 had injected opiates or crack cocaine in the last year, henceforth referred to as the *NTA estimates*.^28^ Of principal interest here is the number of people who have not permanently ceased injecting, which will be a larger group than those that have injected in the last year due to the typical multiple cycles of cessation and relapse before permanent cessation.^29^ We therefore allowed for the number of PWID in our model to be 20%‐60% higher than the number injecting in the last year; these assumptions were explored in sensitivity analyses.

Laboratory reports of antibody‐positive tests were collated by PHE and deduplicated based on date of birth and postcode. Data were available from 1996 to 2016 and were aggregated to 10‐year age groups for analysis. Information on annual numbers treated for chronic HCV infection comes from three sources. Data on treatment sales were used to derive numbers treated with interferon‐based therapy from 2006 to 2011.^6^ Patterns of repeat testing indicative of treatment from sentinel surveillance^30^ were used to predict treatment numbers from 2012 to 2015. From 2016 onwards, numbers treated with new direct‐acting antivirals were provided directly from Bluteq, the system for high‐cost drugs management used by NHS England commissioning.

A more detailed overview of the data is given in Appendix S1.

### 2.2 Model

We constructed a discrete time model with yearly intervals, estimating the number of people entering the PWID group over time, which drives the epidemic. Each year a proportion of current PWID permanently cease injecting and move to an ex‐PWID group. While in the PWID group, individuals are at risk of HCV infection through injecting, although infections may also arise from the noninjecting population.

**Figure 1:**
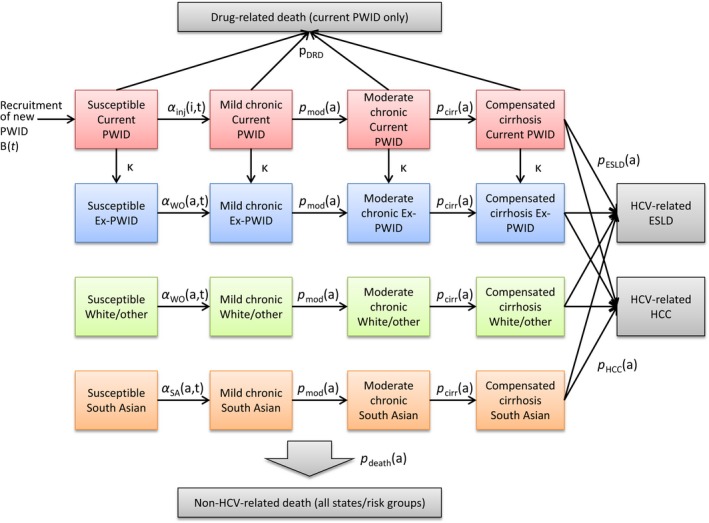
Risk group and disease state structure of the model. For simplicity, transitions to non‐HCV, non‐drug‐related death (which may occur while in any state) are not explicitly shown. Observed data are available for HCV‐related ESLD and HCC; these are not broken down by risk group. State transition probabilities: Parameters are indexed by age (*a*), time (*t*) and injecting duration (*i*). *α*_inj_(*i*,*t*) = infection in people who inject drugs (PWID: those currently injecting or who have temporarily ceased to inject); *α*_WO_(*a*,*t*) = infection in white/other never‐PWID, and ex‐PWID; *α*_SA_(*a*,*t*) = infection in South Asian never‐PWID; κ = permanent injecting cessation; p_DRD_ = drug‐related death; p_death_(*a*) = non‐drug‐related death; p_mod_(*a*) = progression to moderate chronic state; p_cirr_(*a*) = progression to compensated cirrhosis state; p_ESLD_(*a*) = progression to HCV‐related end‐stage liver disease; p_HCC_(*a*) = progression to HCV‐related hepatocellular carcinoma

Chronically infected individuals progress through disease states defined on the basis of a modified HAI score,^2^ *mild chronic*,* moderate chronic* and *compensated cirrhosis*, to the disease endpoints ESLD and HCC (subsequent mortality or other outcomes are not considered). The resulting number of incident ESLD and HCC cases is thus linked to the recruitment of new PWID via a function involving probabilities of infection through injecting drug use and disease progression, plus the infection rate through routes other than injecting drug use. An outline of the risk group and disease stage structure is shown in Figure 1, and further details are available in Appendix S3.

In addition to the risk group/disease state structure described above, the infected population is further compartmentalized into *never* diagnosed, *ever* diagnosed and still infected, and sustained viral response (SVR) states, the latter being the result of successful treatment.^31^ Those in the SVR state may be re‐infected and return to the ever diagnosed state. While in the SVR states, those in mild and moderate chronic disease states do not experience disease progression. Those achieving SVR that have already developed compensated cirrhosis may progress to ESLD or HCC, but with reduced probability.^32^ All individuals continue to progress from their pretreatment disease state if reinfection occurs.

### 2.3 Parameterization and estimation

The key parameters to be estimated are rates of injecting drug use initiation over time, rates of infection in PWID and disease progression probabilities. The model is specified in a Bayesian framework, and parameters are assigned prior distributions on the basis of the information available on them. Posterior distributions are then derived through the combination of the prior distributions and observed data. Probabilities of chronic infection in PWID are estimated via a force of infection model, allowing for an excess risk on initiation and changes over calendar time (Appendix S2). Parameters for disease progression are assigned *informative* prior distributions reflecting the uncertainty of the estimates obtained from the literature, but can be modified by the observed data. Probabilities of permanent cessation of injecting drug use are also assigned informative priors, with 34% stopping within 1 year and the remainder having a mean duration of between 7 and 21 years.^21^ The numbers of individuals with chronic infection moving from undiagnosed to ever diagnosed in the model are based directly on data for the number of new diagnoses in each year. Similarly, the number of individuals moving from diagnosed states to SVR is based on the derived/assumed numbers treated, and the SVR rate. Probabilities of treatment, conditional on having been diagnosed, are assumed equal across age groups, risk groups and disease stages (these assumptions are explored in sensitivity analysis). Probabilities of SVR under interferon‐based therapies are assigned fixed values based on published estimates,^8^ and new therapies assigned a fixed 90% intention‐to‐treat probability of SVR. The infection rate via routes other than injecting drug use, annual probabilities of mortality and post‐SVR risk ratios for developing ESLD/HCC are assumed to be known (see Appendix S4).

We derived the posterior distribution of parameters through Markov Chain Monte Carlo methods on the basis of 20 000 samples run in two parallel chains, following a “burn‐in” period of 2000 iterations. Posterior distributions of parameters and functions (such as predicted chronic prevalence) were summarized via their medians (point estimates) and 2.5th and 97.5th percentiles to form 95% credible intervals (CrI). Further details of the model implementation are given in Appendix S5.

## 3 RESULTS

We began by describing estimates of the key parameters and model fit. A key decision was to restrict analysis to the 2011‐2016 period of HES data in the base model. We present results on key parameters from this model, as well as those obtained from using the full range of data (2004‐2016) and earlier data (2004‐2010). Prevalence estimates and other derived quantities are then described for the base model. Finally, we explored the impact of different assumptions on estimated prevalence in sensitivity analyses.

### 3.1 Parameter estimates and model fit

**Figure 2:**
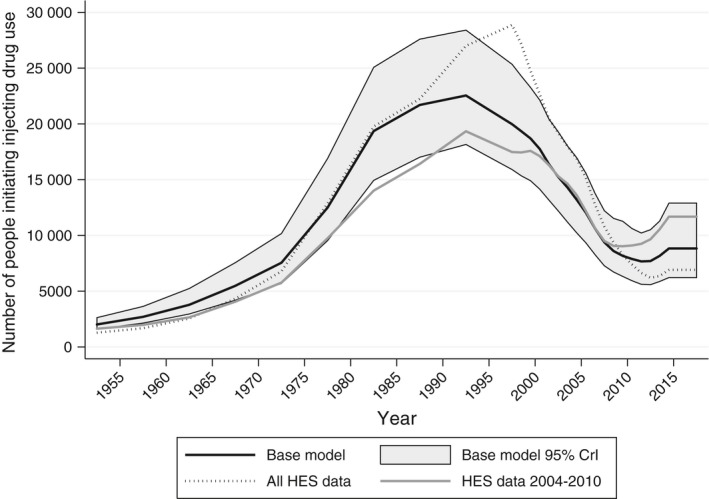
Number of people initiating injecting drug use over time. Number of people initiating injecting drug use over time under the base model, posterior medians and 95% credible intervals (CrI). Posterior medians under models using all HES data (2004‐2016) and data for 2004‐2010 are also displayed

Figure 2 shows the estimated number of people initiating injecting drug use over time. Under the base model, we estimated that the number of people initiating injecting drug was below 5000 per year until the early 1960s, increased rapidly in the 1970s and peaked at over 20 000 per year in the late 1980s. Numbers then started to fall in the 1990s until they are below 10 000 per year in the late 2000s. When including HES data from all years, the estimated peak is later, but higher. Under the 2004‐2010 HES data, numbers are generally lower, but again with a slightly later peak.

**Figure 3:**
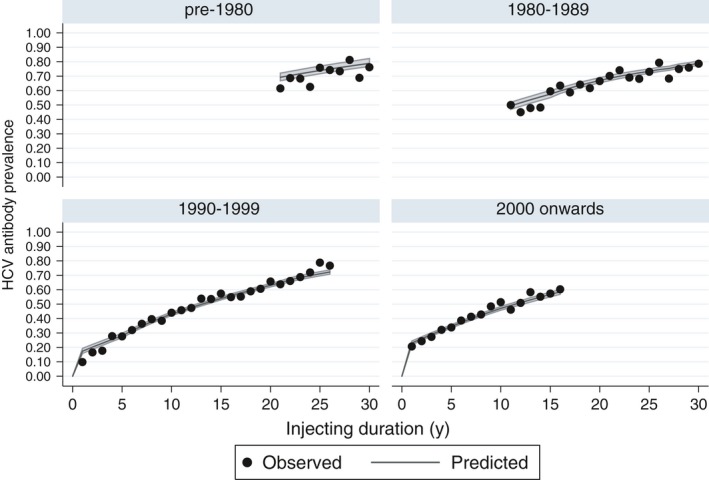
Observed and predicted HCV antibody prevalence in people who inject drugs. Data from the Unlinked Anonymous Monitoring survey 2000‐2016 split into four groups of year started injecting. Posterior medians and 95% credible intervals for HCV prevalence under the base model

The estimated annual rate of chronic infection after the first year of injecting is 3.3 per 100 person‐years (95% CrI: 2.1‐3.9) prior to 1980, 4.2 (95% CrI: 3.7‐5.0) from 1980 to 1985, then fell to 2.3 (95% CrI: 2.1‐2.5) in 1995 to 2000 before rising slightly to 3.2 (95% CrI: 3.0‐3.6) from 2015 onwards. The estimated hazard ratio for the first year versus subsequent injecting career is 6.27 (95% CrI: 5.84‐6.85), with 19% of those injecting for one year testing positive for HCV antibodies in the UAM data, rising to over 40% in those injecting for 8 years or more. Figure 3 shows observed HCV antibody prevalence in the UAM data and that predicted under the model. The model captures the relationship between injecting duration and proportion infected over time well, although credible intervals are very narrow and there is some over‐dispersion. Estimates were similar when modelling different periods of HES data.

**Figure 4:**
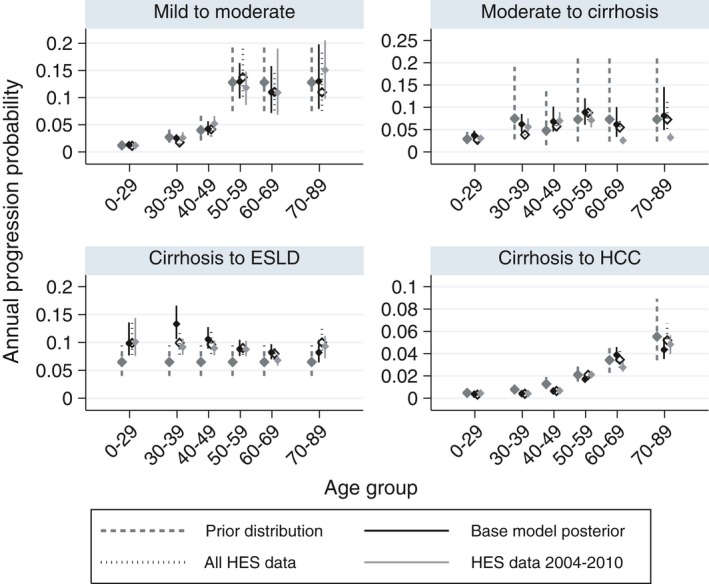
Estimated disease progression probabilities. Posterior medians and 95% credible intervals for age‐specific posterior progression probabilities under the base model, and fitting to different periods of HES data. Prior distributions are also shown, with medians and 95% probability intervals. *Y*‐axis scales vary across subplots

Figure 4 shows posterior estimates of disease progression probabilities. Estimates from the base model were generally close to the prior values, although posterior probabilities of progression from cirrhosis to ESLD were higher than those specified by the prior, in particular for younger ages (0‐29 and 20‐39). The results indicate that for a 20‐year‐old, the median duration between chronic infection and developing compensated cirrhosis (provided death does not occur) is 37 years (95% CrI: 34‐40), and for ESLD or HCC, 46 years (95% CrI: 43‐50). For a 30‐year‐old, the median duration between infection and compensated cirrhosis is 29 years (95% CrI: 26‐33), and for ESLD or HCC, 38 years (95% CrI: 35‐42). Results followed a broadly similar pattern when fitting to the full series of HES data, but with slightly longer time from chronic infection to severe disease.

**Figure 5:**
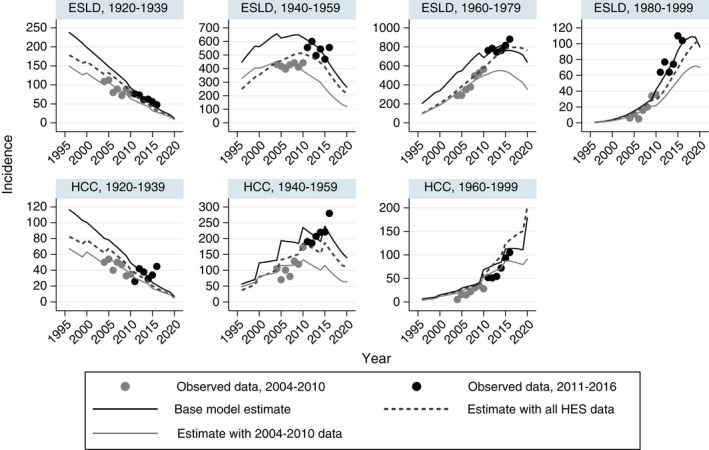
Observed and predicted end‐stage liver disease and hepatocellular carcinoma. Results by 20‐y birth cohorts under the base model and using different periods of HES data. *Y*‐axis scales vary across sub‐plots. There are fewer than five cases of HCC in those born from 1980 to 1999, and this group is pooled with the 1960‐1979 birth cohort. Data source: Hospital Episode Statistics (HES), NHS Digital for England. Produced by Public Health England

Figure 5 shows observed and predicted HCV‐related ELSD and HCC over time, by birth cohort. We estimated a steady decline in ESLD/HCC in those born prior to 1940, a rise and decline in those born between 1940 and 1959, and rising incidence up to 2015 in those born after 1960. We were unable to obtain a good fit to the full series of HES data (2004‐2016). ESLD and HCC cases rise sharply in some birth cohorts, in particular around 2011, which is implausible for a disease with long incubation time and time‐invariant disease progression. When restricting analysis to the 2011‐2016 period, a better fit was obtained to these data, but the model produced over‐estimates for the 2004‐2010 period. Conversely, modelling the 2004‐2010 data alone resulted in under‐estimates for 2011‐2016.

### 3.2 Prevalence estimates and predictions

We estimated that the number of PWID aged 15‐64 in 2011 was 135 000 (95% CrI: 95 000‐178 000, rounded to nearest 1000), around 44% larger than 2011/12 estimates of the number of people who had injected in the last year used in the model. The number of PWID is estimated to have peaked in 1998 at 178 000 (95% CrI: 140 000‐217 000) and declined to 123 000 (95% CrI: 84 000‐166 000) in 2015. The incidence of chronic infection is estimated to have been below 2000 per year prior to 1970, then risen to a peak of 7100 (95% CrI: 6000‐8400) per year in the early 1990s, then declined to 4300 (95% CrI: 3400‐5400) per year in 2015.

Table 1 shows estimates of current and ex‐PWID and numbers with chronic HCV infection, by age group and overall, for 2005 and 2015. In 2015, we estimated that there were 121 100 (95% CrI: 83 300‐167 800) PWID and 48,100 (95% CrI: 30 500‐71 200) with chronic infection (40%, 95% CrI: 36%‐44%). The number of PWID declined from 2005, but the proportion with chronic infection stayed stable. The estimated number of ex‐PWID is much higher than the number of PWID, at 513 200 (95% CrI: 415 200‐598 700), but only 16% (95% CrI: 15%‐17%) with chronic infection, giving 80,900 (95% CrI: 64 400‐95 700) ex‐PWID with chronic infection. The lower proportion infected in ex‐PWID is due to ex‐PWID tending to have shorter injecting duration (time at risk) than those still injecting, and in particular roughly one‐third ceasing to inject within one year. The proportion with chronic infection rises with age in both current and ex‐PWID, due to longer average durations of injecting. The average age of both current and ex‐PWID is estimated to have increased. We estimated that in 2005, 26% of PWID were over 40, increasing to 38% in 2015, and the proportion of ex‐PWID aged over 50 was estimated to have increased from 27% in 2005 to 45% in 2015.

**Table 1:** Estimated risk group sizes and chronic infections, 2005 and 2015

Age group	Group size	Chronic infection	% infected
2005			
PWID			
0‐29	64 900 (56 100‐74 100)	18 200 (15 500‐20 800)	28% (27‐29%)
30‐39	56 800 (42 600‐73 800)	24 000 (17 300‐32 300)	42% (40‐44%)
40‐49	30 600 (19 000‐47000)	16 700 (10 000‐26 400)	55% (52‐56%)
50‐59	9500 (4850‐17 000)	5900 (2900‐10 800)	62% (59‐64%)
60+	2000 (880‐4350)	1350 (590‐2950)	67% (64‐70%)
Total	164 200 (123 600‐213 300)	66 200 (46 400‐92 400)	40% (37‐44%)
Permanently ceased injecting			
0‐29	68 600 (56 500‐84 800)	8500 (6100‐11 300)	12% (11‐14%)
30‐39	134 400 (108 300‐164 300)	27300 (20 400‐34 500)	20% (19‐21%)
40‐49	141 700 (112500‐176 300)	35 200 (26 700‐43 100)	25% (23‐26%)
50‐59	80 200 (64 100‐101 800)	19 300 (15 100‐24 100)	24% (21‐26%)
60+	46 900 (37 500‐56 800)	8700 (7000‐11200)	19% (15‐22%)
Total	474 700 (380 200‐568 700)	99 400 (75 800‐119 400)	21% (20‐22%)
Never injected drugs^a^			
South Asian ethnicity	2 641 000	8500	0.32%
White/other ethnicity	4 7500 000	10 500	0.022%
All risk groups			
Total	53 910 000	179 000 (161 000‐198 000)	0.35% (0.32‐0.39%)
2015			
Current PWID			
0‐29	39 000 (30 300‐51 500)	10 800 (8600‐14 600)	28% (26‐30%)
30‐39	36 100 (25 300‐47 900)	13 700 (9300‐18 600)	38% (36‐39%)
40‐49	29 000 (17 100‐45 300)	13 600 (7700‐22100)	47% (45‐50%)
50‐59	13 600 (6500‐25 300)	7500 (3500‐14600)	56% (54‐59%)
60+	3750 (1450‐8500)	2350 (870‐5400)	61% (59‐64%)
Total	121 100 (83 300‐167 800)	48 100 (30 500‐71 200)	40% (36‐44%)
Permanently ceased injecting			
0‐29	37 100 (28 800‐47 900)	4500 (3100‐6300)	12% (11‐14%)
30‐39	92 600 (75 200‐114 100)	13 000 (9900‐16 600)	14% (13‐15%)
40‐49	151 000 (122 800‐180 500)	25 100 (20 000‐30 900)	17% (16‐18%)
50‐59	135 600 (109 500‐168 900)	24700 (19 600‐31 200)	18% (17‐20%)
60+	92 700 (76 200‐117 400)	13 100 (9700‐17 400)	14% (12‐17%)
Total	513 200 (415 200‐598 700)	80 900 (64 400‐95 700)	16% (15‐17%)
Never injected drugs^a^			
South Asian ethnicity	3 331 000	8300	0.25%
White/other ethnicity	49 950 000	8200	0.016%
*All risk groups*			
Total	50 770 000	143 000 (123 000‐161 000)	0.27% (0.23‐0.30%)

Posterior medians and 95% credible intervals for the number of PWID and ex‐PWID^b^, and number (%) with chronic infection, by age group. Total numbers of those who have never injected drugs are also given^a^.

a Statistical uncertainty is not fully incorporated in estimated infections in those who have never injected drug; credible intervals are therefore not displayed.

b **PWID** are defined as those currently injecting or who have temporarily ceased to inject; **ex‐PWID** have injected in the past, but now permanently ceased.

In total, we estimated that there were 143,000 (95% CrI: 123 000‐161 000) people with chronic HCV infection in 2015, 45 000 of which have had a positive HCV test reported to PHE (a lower bound on the total number diagnosed as reporting is not complete). We estimated that 179 000 (95% CrI: 161 000‐198 000) people had chronic infection in 2005, 36 000 more than in 2015.

We assumed that numbers treated with direct‐acting antivirals will increase from around 11 500 per year in 2017 to 15 000 per year in 2020, in line with the planned rollout of treatment by NHS England at the time of writing. As a result, we predict that the number of people with chronic infection will decrease to 113 400 (95% CrI: 94 900‐132 400) by the end of 2018 and to 89 500 (95% CrI: 71 300‐108 600) by the end of 2020, reductions of around 20% and 37%, respectively, compared to 2015. The proportion of infected PWID was predicted to decrease to 28% (95% CrI: 25%‐32%) in 2020, and incident ESLD/HCC was predicted to decrease by around 24%, from 1770 (95% CrI: 1610‐1880) in 2015 to 1350 (95% CrI: 1160‐1510) in 2020. Under the assumption that diagnosis and DAA treatment are more likely in those with cirrhosis (with a median time to diagnosis of less than 2 years and median time to treatment <2 years), the reduction is closer to one‐half.

### 3.3 Sensitivity analyses

We tested a variety of alternative model formulations to assess the sensitivity of results to the assumptions, including using different periods of HES data, using data for a single disease endpoint (ESLD or HCC), and alternative numbers of PWID and average injecting durations. We also explored the potential impact of imperfect reporting of HCV in HES data, such that true numbers are 42% higher.^33^ Table 2 summarizes results under different models in terms of the main quantities of interest, the total number of chronic infections in 2005 and 2015.

**Table 2:** Estimated chronic prevalence with 95% credible intervals for 2005 and 2015 under alternative model formulations and sensitivity analyses

Model	2005 prevalence	2015 prevalence
Base model	179 000 (161 000‐198 000)	143 000 (123 000‐161 000)
All HES data (2004‐2016)	192 400 (174 200‐214 100)	164 800 (144 300‐188 100)
HES data 2004‐2010	146 200 (134 100‐159 600)	120 700 (107 000‐136 000)
All HES data, no NTA data	227 000 (193 600‐248 500)	218 200 (175 500‐252 800)
ESLD only (all years), no NTA data	265 900 (239 600‐289 000)	263 300 (231 800‐290 300)
HCC only (all years), no NTA data	189 400 (171 900‐206 400)	150 900 (129 400‐169 600)
True HES data 42% higher	218 300 (195 200‐239 600)	169 000 (147 300‐193 000)
HES data 42% higher, HCC only	190 300 (171 800‐216 400)	150 300 (130 500‐173 700)
HES 42% higher, stronger PWID prior	195 700 (178 200‐214 400)	139 800 (121 700‐157 300)
Fixed 100 000 PWID, mean injecting 20 y	158 100 (152 200‐163 700)	105 500 (99 700‐110 100)
Fixed 100 000 PWID, mean injecting 10 y	237 000 (210 400‐254 700)	187 700 (165 800‐202900)
Fixed 200 000 PWID, mean injecting 20 y	199 400 (188700‐209400)	174 800 (166 300‐184 000)
Fixed 200 000 PWID, mean injecting 10 y	248 200 (226 400‐268 300)	241 900 (223 600‐263 100)

A key decision was to restrict analysis to the 2011‐2016 period of HES data. The estimated number of chronic infections in 2015 was lower based on the 2004‐2010 HES data, but higher when using the full 2004‐2016 data.

The NTA estimate of the number of PWID influences the number of people with chronic infection, given the risk of infection in PWID and cessation rate. If this information is omitted, the number of chronic infections in 2015 was estimated to be 218 200 (175 500‐252 800). This is largely driven by the data on ESLD: when modelling data on HCC alone, estimates were comparable to the base model, but were far higher when modelling data on ESLD alone. This is due to the rapid increase in ESLD in those born since 1980 (Figure 5). Without the constraint on the number of PWID, the trend in younger individuals developing ESLD suggests that the injecting drug use epidemic has actually worsened since the 1990s with a rapidly expanding population of HCV‐infected individuals.

A key assumption of our model is complete reporting of HCV‐related severe liver disease; if the true incidence is higher, this pushes prevalence upwards. The impact is smaller if HCC alone is considered, and placing a strong prior on the size of the PWID population at 20% higher than the reported estimate results in a similar estimate of 2015 prevalence, with disease progression probabilities shifted upwards to resolve the conflict in the data.

We examined the impact of assuming fixed values for the number of PWID in 2011 of 100 000 (slightly higher than the NTA estimate) and 200 000, and of fixing the mean injecting duration at 10 and 20 years. A higher number of PWID imply higher prevalence; a longer injecting duration implies comparably fewer ex‐PWID and lower overall prevalence. The fit to the HES data was generally comparable under different assumptions, but a larger PWID population gave a better fit to the ESLD data, while a smaller population gave a better fit to the HCC data.

## 4 DISCUSSION

We have described a model for HCV in England that makes use of routinely collected data and can be used to guide public health planning and monitor progress towards the goal of elimination. We estimated 143 800 chronic infections (95% CrI: 123 000‐161 000) in 2015. The model indicated a peak in prevalence around 2005 and subsequent fall, which is associated with a decline in the number of people initiating injecting drug use, and some success of interferon‐based treatments over this period.

Rapid reductions in prevalence are expected due to the expanding delivery of new treatments from 2015 to 2020, with 89 500 (95% CrI: 71 300‐108 600) chronic infections predicted by the end of 2020. Reductions in severe HCV‐related liver disease are expected to be smaller, due to an ageing‐infected population at increasing risk of developing severe liver disease. We also assume equal access to treatment across disease stages, which is conservative. However, higher rates of treatment in those with cirrhosis still do not result in very low levels of severe HCV‐related liver disease, due to the possibilities of rapid disease progression before treatment and continued disease progression post‐SVR.^6^ Nevertheless, any reduction would still be promising, given the projected rise well beyond 2020 in the absence of treatment scale‐up.^6^ The impact of DAAs on severe HCV‐related liver disease will be further explored as more detailed treatment and outcomes data, in particular for those with cirrhosis, become available. As of 2017, PCR testing is being undertaken in the UAM study, which will also allow a more direct assessment of chronic prevalence and impact of treatment in this group.

We estimated around 45 000 individuals with diagnosed chronic infection had been reported to PHE and were still living with chronic infection in 2015. Although this may be a lower bound on the total number diagnosed due to under‐reporting, there is a concern that the number of diagnosed individuals available to be treated may not keep pace with planned increases in treatment.

The model developed here provides an understanding of how routinely collected data are related to the underlying epidemic, and allows assessment of progress towards targets that are not directly measurable. For instance, we predicted that the number of chronic infections would be reduced by 37% by 2020 (compared to 2015) under current treatment plans. This cannot be measured directly, although the predicted reductions of 29% chronic prevalence in PWID and 24% in incident ESLD/HCC that should be associated with such a decrease can be assessed through UAM and HES data, respectively. Changes in HCV‐related mortality will broadly follow that of HCV‐related ESLD/HCC; therefore, meeting the WHO target of a 10% decrease in HCV‐related mortality by 2020 is likely for England.^10^

The WHO target of a 30% reduction in incidence is difficult to monitor, as many new infections go undetected and diagnosis may occur many years after infection. Markers of recent infection such as avidity testing^34^ can only reliably detect a large reduction in incidence.^35^ Our model can be used to estimate incident infection over time, via changes in observed prevalence in the UAM survey of PWID. A full understanding of the process giving rise to new infections would need to incorporate the dynamic effects of treatment as prevention and harm reduction interventions in PWID.^36^

Estimating the number of people living with chronic HCV infection is inherently difficult, due to the uncertainties in the size of PWID and ex‐PWID populations. Further work is required to better understand the size of the at‐risk population and those with a history of past injecting risk. Work is in progress to develop models using overdose mortality and drug treatment data to estimate opiate use. There is a particular lack of knowledge around individuals who cease injecting after a short period, who may be at lower risk of infection and never attend services in which they might be observed.^37^ Our analysis assumes that 34% of those who start injecting drugs permanently cease within 1 year, although the impact of different assumed proportions and levels of risk on overall prevalence was minimal, with only the estimated number of ex‐PWID changing materially.

We chose to base inferences on more recent HES data (2011‐2016), which resulted in higher estimates of HCV‐related ESLD/HCC than those observed for the 2004‐2010 period. The rapid increase in observed numbers of ESLD/HCC around 2010‐2011 may be explained by changes in HCV reporting, which may have improved generally after laboratory notification of positive HCV tests becoming mandatory in 2010. Other factors explaining the observed patterns may include changes in levels of alcohol use, which accelerates disease progression, or changes in the average age at infection over time.^2^, ^38^

Under‐reporting of HCV in HES data is likely, and we explored the impact of the true incidence of HCV‐related ESLD and HCC being 42% higher.^33^ Under the base assumptions, this increases estimates of chronic prevalence, although 2015 results are comparable to the base model if stronger prior information is placed on the number of PWID. With a smaller PWID population, disease progression rates are pushed upwards.

Our model incorporates uncertainty in the key parameters, but also includes a number of strong assumptions. The associated uncertainty of our estimates is therefore likely to be underestimated. Future modelling will aim to fully account for the statistical uncertainty of all parameters.

Previous multi‐parameter evidence synthesis (MPES) models provided an estimate of 160 000 chronic infections in 2005, which has been widely used for planning purposes.^19^, ^35^ Our new results are consistent with previous MPES estimates for 2005, with similar age and risk group distributions; although providing a slightly higher estimate of people with chronic infection of 179 000, the credible intervals of the estimates overlap substantially. The Polaris Observatory estimated that in the UK there were 189 000 chronic infections in 2015.^39^ Their modelling uses previously published estimates of prevalence and expert opinion to derive a starting point for the infected population in 2005 and applies a forward projection model using estimates of disease progression^6^, ^38^ to extrapolate. A similar, but more detailed approach has been used in the United States.^13^ In comparison, we make no assumptions about starting prevalence and incorporate a wealth of surveillance data within a formal statistical model.

Our estimates indicate that the size of the PWID population was around 44% larger than 2011/2012 estimates of the number of people who had injected in the last year.^28^ This might be explained by the difference in definitions, as prior to permanent cessation PWID may have multiple, sustained periods of cessation.^29^ An earlier study estimated the number of opiate and injecting drug users via back calculation from drug‐related deaths,^4^ which gave lower estimates of current and past opiate and injecting use than indicated here. Their study estimated an increase in opiate use in the 1970s, generally stable in the 1980s, and subsequent increase in the early 1990s.

A number of studies have used back calculation to predict the future course of the HCV epidemic.^6^, ^15^, ^17^, ^20^ Such modelling typically estimates the historical pattern of infections, but not the underlying population at risk. Our study builds on this approach, simultaneously estimating the process of initiating injecting drug use, rates of HCV infection and disease progression within a statistical model. This general framework could be applied to other countries and incorporate different data sources on disease endpoints, serosurveillance and injecting drug use as available, with the aim of constructing a picture of the HCV epidemic that is consistent with all relevant sources of information.

In conclusion, our analytic approach provides the template for revising and updating estimates of chronic HCV in England and elsewhere, and the method for evaluating HCV treatment scale‐up. Our model provides an estimate of between 123 000 and 161 000 chronic infections in 2015, which would be a suitable range to consider for planning purposes, although the possibility of higher prevalence cannot be discounted. We predict that direct‐acting antiviral treatments will lead to a substantial reduction in HCV prevalence in the short term and meet WHO targets for HCV‐related mortality in 2020. However, diagnosis rates and engaging patients in care will require substantial improvement for elimination of HCV as a major public health threat to be a viable prospect in the next decade.
