# Supplementary material for: Monitoring the hepatitis C epidemic in England and evaluating intervention scale‐up using routinely collected data
Source: J Viral Hepat. 2019 Feb 28;26(5):541–51. doi: 10.1111/jvh.13063 (PMC6518935; doi:10.1111/jvh.13063)
Supplement: Supplementary file 1 [file JVH-26-541-s001.docx]

## Appendix

## S1. Data sources

### HES data

Hospital episode statistics (HES) data on ESLD and HCC are used as disease endpoints. Records are available from the beginning of 1997 to the end of 2016. A unique HES ID number was used to identify individuals in the data. ESLD was defined by ICD10 codes for ascites (R18), bleeding oesophageal varices (I850); hepato-renal syndrome (K767), hepatic encephalopathy or hepatic failure (K704, K720, K721, K729). HCC was defined by ICD10 code C22.0. Hospital episodes were defined as HCV-related if any HES-recorded episode in the patient’s history, before or after ESLD/HCC occurred, included a diagnosis code for HCV (B171 or B182). This approach is employed to minimise under-reporting of HCV, which may have been recorded in a previous episode, or the patient tested subsequent to first presentation with ESLD/HCC. However, the majority of those with an HCV diagnosis are coded as such at the time of the ESLD/HCC episode.

First episodes of ESLD or HCC, whichever occurred first in an individual, are taken as incident cases of the disease endpoints. A case of HCV-related ESLD or HCC is defined as a ‘first’ episode if no previous episodes of ESLD or HCC for that individual are found in at least the previous five years (less than 1% of ESLD/HCC episodes are estimated to have had a previous episode more than five years earlier). Data from 2004 onwards are aggregated by 10-year birth cohorts prior to analysis, and are summarised in Table S1, further aggregated into 20-year birth cohorts. There were fewer than 5 cases of HCC in those born from 1980-1999; this group is pooled with the 1960-1979 birth cohort to avoid any issues with small cell sizes.

Table S1. Incidence of HCV-related end stage liver disease (ESLD) and hepatocellular carcinoma (HCC) over time, by 20-year birth cohorts.

| Year | ESLD | | | | HCC | | |
| --- | --- | --- | --- | --- | --- | --- | --- |
|  | pre-1940 | 1940-59 | 1960-79 | 1980-99 | pre-1940 | 1940-59 | 1960-99 |
| 2004 | 110 | 430 | 288 | 6 | 53 | 105 | 5 |
| 2005 | 120 | 418 | 290 | 11 | 54 | 70 | 16 |
| 2006 | 84 | 395 | 352 | 5 | 40 | 101 | 15 |
| 2007 | 92 | 428 | 377 | 16 | 50 | 81 | 22 |
| 2008 | 72 | 443 | 494 | 20 | 47 | 129 | 30 |
| 2009 | 89 | 411 | 528 | 34 | 33 | 119 | 35 |
| 2010 | 78 | 442 | 564 | 36 | 35 | 173 | 28 |
| 2011 | 76 | 556 | 760 | 64 | 26 | 190 | 52 |
| 2012 | 73 | 600 | 784 | 77 | 42 | 186 | 51 |
| 2013 | 61 | 496 | 745 | 64 | 38 | 207 | 54 |
| 2014 | 62 | 544 | 760 | 74 | 29 | 220 | 72 |
| 2015 | 56 | 469 | 817 | 110 | 34 | 221 | 93 |
| 2016 | 48 | 555 | 882 | 104 | 45 | 280 | 105 |

Data source: Hospital Episode Statistics (HES), NHS Digital for England.

Produced by Public Health England.

### Unlinked Anonymous Monitoring (UAM) survey of people who inject drugs

The UAM survey of people who inject drugs samples from harm reduction and treatment services across England ^1^. Respondents voluntarily and anonymously provide a serological sample, using dried blood spots as of 2010 and oral fluid samples previously. The sample is tested for antibodies to HCV, and participants complete a brief questionnaire on demographics and risk behaviour, in particular their current age and age at first injecting drug use, allowing calculation of their injecting duration. The information on time at risk and proportion with HCV antibodies is used to estimate the rate of infection in PWID (see section A2).

In the analyses here only those that have injected in the last year are considered. Data are used from the years 2000 to 2016, comprising 32,564 individuals with complete information on injecting duration and HCV test status. Table S2 shows a summary of the data, grouped by injecting duration and period started injecting. This table highlights the available information for estimating the force of infection over time, which results from the increase in prevalence with exposure time.

Table S2. Observed HCV antibody prevalence by injecting duration and year started injecting, data from the UAM survey of people who inject drugs (2000-2015).

| Injecting duration (years) | Year started injecting | | | |
| --- | --- | --- | --- | --- |
|  | pre-1980 | 1980-1989 | 1990-1999 | 2000 onwards |
| ≤1 |  |  | 44/449 (9.8%) | 577/2800 (20.6%) |
| 2 |  |  | 79/477 (16.6%) | 364/1500 (24.3%) |
| 3-4 |  |  | 259/1116 (23.2%) | 657/2224 (29.5%) |
| 5-6 |  |  | 433/1450 (29.9%) | 646/1785 (36.2%) |
| 7-9 |  |  | 616/1619 (38.0%) | 538/1281 (42.0%) |
| 9-11 |  | 40/80 (50.0%) | 1169/2727 (42.9%) | 609/1246 (48.9%) |
| 12-14 |  | 259/548 (47.3%) | 1159/2249 (51.5%) | 430/791 (54.4%) |
| 15-19 |  | 1025/1668 (61.5%) | 1659/2902 (57.2%) | 133/228 (58.3%) |
| 20-24 | 139/212 (65.6%) | 1082/1559 (69.4%) | 805/1213 (66.4%) |  |
| 25-29 | 281/378 (74.3%) | 668/897 (74.5%) | 90/115 (78.3%) |  |
| ≥30 | 545/681 (80.0%) | 302/369 (81.8%) |  |  |

### Diagnosis data

Laboratory reports of positive HCV antibody tests in England are reported to Public Health England ^2^. Notification of positive HCV tests became mandatory in 2010, although reporting is likely to still be incomplete. Repeat tests are de-duplicated based on date of birth and postcode to obtain the first known positive test date for each individual. Data are aggregated by year and 10-year age group, and summarised in Table S3.

Table S3. First reported positive HCV antibody tests in England from laboratories reporting to Public Health England, by age group (1996-2016).

| Year | Age group | | | | |
| --- | --- | --- | --- | --- | --- |
|  | 0-29 | 30-39 | 40-49 | 50-59 | ≥60 |
| 1996 | 565 | 709 | 424 | 109 | 139 |
| 1997 | 692 | 882 | 506 | 111 | 154 |
| 1998 | 1,151 | 1,411 | 767 | 230 | 232 |
| 1999 | 1,437 | 1,677 | 941 | 269 | 262 |
| 2000 | 1,411 | 1,617 | 902 | 282 | 242 |
| 2001 | 1,220 | 1,536 | 852 | 339 | 258 |
| 2002 | 1,431 | 1,864 | 1,025 | 369 | 256 |
| 2003 | 1,662 | 2,131 | 1,255 | 451 | 334 |
| 2004 | 1,835 | 2,433 | 1,558 | 693 | 379 |
| 2005 | 1,766 | 2,227 | 1,523 | 750 | 356 |
| 2006 | 1,750 | 2,442 | 1,779 | 800 | 409 |
| 2007 | 1,724 | 2,639 | 2,122 | 1,034 | 479 |
| 2008 | 1,804 | 2,742 | 2,272 | 1,122 | 499 |
| 2009 | 1,738 | 2,756 | 2,392 | 1,182 | 483 |
| 2010 | 1,427 | 2,441 | 2,149 | 1,247 | 566 |
| 2011 | 1,753 | 2,975 | 2,759 | 1,598 | 780 |
| 2012 | 1,829 | 3,338 | 3,094 | 1,820 | 929 |
| 2013 | 1,779 | 3,363 | 3,193 | 1,906 | 925 |
| 2014 | 1,699 | 3,073 | 2,843 | 2,030 | 1,140 |
| 2015 | 1,521 | 2,926 | 2,690 | 2,086 | 1,203 |
| 2016 | 1,532 | 3,071 | 2,759 | 2,030 | 1,353 |

### Treatment data

IMS data on treatment prescribing are used to derive numbers treated with interferon-based therapy from 2006-2011 ^3^. Patterns of repeat testing indicative of treatment from sentinel surveillance ^4^ were used to predict numbers on treatment from 2012 to 2015. Sentinel surveillance does not cover the whole population, and are estimates of treatment activity, which may under- or over-estimate the true number treated. Predicted numbers of people treated in England are therefore obtained by taking the average ratio of IMS:sentinel data for 2006-2011 and using this to scale up the estimates from sentinel surveillance for 2012-2015. Figure S1 shows the IMS treatment and sentinel surveillance data, and resulting predicted numbers treated.

Figure S1. IMS data on HCV treatment and information on treatment from participating sentinel surveillance laboratories, with resulting predicted numbers treated.

From 2016 onwards numbers treated with new direct acting antivirals were available directly from NHS England from the Bluteq system, the system for high cost drugs management process used by NHS England commissioning. Data are available for the financial years 2016/17 and 2017/18, taken in this work to represent the calendar years 2016 and 2017 as the timescale used in the model used here is in terms of calendar years.

Table S4 shows the resulting numbers treated used for analysis, using the IMS prescribing data, 2012-2015 estimates, Bluteq data and assumed treatment rollout.

Table S4. Data, estimates and data on HCV treatment, and future assumptions.

| Data | Year | Number treated |
| --- | --- | --- |
| IMS prescribing data on IFN-based treatment | 2006 | 3270 |
|  | 2007 | 4059 |
|  | 2008 | 4561 |
|  | 2009 | 5046 |
|  | 2010 | 5360 |
|  | 2011 | 5160 |
| Estimated IFN-based treatment (Sentinel Surveillance) | 2012 | 4926 |
|  | 2013 | 4996 |
|  | 2014 | 4690 |
|  | 2015 | 4181 |
| DAA treatment data (Bluteq) | 2016 | 9440 |
|  | 2017 | 11557 |
| Assumed DAA treatment rollout | 2018 | 13000 |
|  | 2019 | 14000 |
|  | 2022 | 15000 |

### Population data

Denominators for total populations of white/other and South Asian ethnicities over time were based on a combination of ONS census estimates for 2001 and 2011 and the ETHPOP database of ethnicity-specific population projections from 2011 to 2030 (<http://www.ethpop.org/>). As ONS data are not available according to age, ethnic group and geographic area, and the starting data for the model is 1930, denominators were derived from age- and ethnic group-specific log-linear trends estimated from the data. Figure S2 shows the data sources used and resulting derived trends.

Figure S2. ONS census data and Ethpop estimates of population size for South Asian and other ethnicities, and modelled trend.

The log-linear model is clearly an over-simplification of how population sizes have changed over time; however, this part of the model is used only to provide a denominator for the background rate of infections in those who have never injected drugs, with people who have ever injected drugs being the main risk group.

## S2. Force of infection model for HCV prevalence in people who inject drugs

We estimated the force of infection (FOI), the rate at which susceptible individuals acquire infection, for HCV infection using the UAM data. FOI models use age-specific seroprevalence data, where “age” in this context is the duration of injecting risk, to estimate the FOI via the relationship between time at risk and prevalence ^5^. Where data are available at multiple time points, as in the UAM, independent age (injecting duration) and temporal effects may be estimated ^6^. Time and injecting duration effects are assumed to be independent, such that injecting duration-specific hazard ratios are constant over time. Interactions between injecting duration and time can only be identified within the range of the survey data, and were not found to provide a substantial improvement in model fit.

The FOI model is integrated within the back-calculation model, such that parameters are estimated simultaneously with the other parts of the model. The FOI is assumed constant in long-term injectors apart from temporal variation, indexed via time *t*, although new initiates have a different level of risk. Indexing injecting duration by *x*={0,1} for long-term and recent injectors, the FOI is thus specified as:

$\lambda_{x,t}=exp(\beta_{t}+\beta_{x}x)$,

where $\beta_{t}$ is the baseline log FOI at time *t* and $\beta_{x}$ the hazard ratio for the first year of injecting. The proportion susceptible to infection after injecting for *a* years at time *t* is then defined by

$S_{a,t}=\exp\left( -\int_{0}^{a} \lambda_{u_{(0,1)},t-u}du \right)$,

where $u_{(0,1)}$ is a step function taking the value 1 if *u*≤1 and zero otherwise to index recent initiates and long-term PWID as defined for $\lambda_{x,t}$.

The log baseline FOI $\beta_{t}$ is modelled via a piecewise constant function with pre-1980 and 5-year intervals up to 2010, then 2010 onwards. The integral above therefore has the form of a sum of FOIs: that for the first year of injecting, and subsequent years of injecting within each time band if *a*>1. As *a* only takes integer values, this simplifies to

$$S_{a,t}=\exp\left( -\lambda_{1,t-u}-\sum_{u=2}^{a} \lambda_{0,t-u} \right)$$

We assume that 24% of infections clear spontaneously (although leaving detectable antibodies) and 76% develop into chronic infections, giving the *chronic* FOI

$\lambda_{x,t}^{c}={0.76 \lambda}_{x,t}$.

This is converted to an annual probability of chronic infection

$\alpha_{x,t}=1-exp(-\lambda_{x,t}^{c})$,

which is used in the back calculation model for the annual probability of chronic infection in susceptible PWID. The probability of infection in the first year of injecting, $\alpha_{1,t}$, is applied to new PWID, and long term PWID have annual probability $\alpha_{0,t}$ subsequently.

Short-term PWID that cease injecting within one year of starting, are assumed to be at risk for one-third of a year. The definition of injecting duration is based on age at first use and age at cessation in completed years; a uniform distribution across calendar years for starting and stopping injecting would imply a mean of one-third in those with the same starting and stopping age. However, applying this reasoning to those who stop injecting after a short period is still an assumption, as the distribution of time at risk in this specific group has not been studied.

We further assume that during this period, short-term PWID are subject to the same first-year FOI as those that go on to inject for longer periods This assumption is made in the absence of any information on the risk behaviour of those that cease to inject within a short period of time. The probability of chronic infection in short-term PWID is therefore defined as:

$\alpha_{t}^{S}=1-exp(-\frac{1}{3}\lambda_{1,t}^{c})$.

In any case, the impact of these assumptions on estimates of HCV prevalence is small, due to the relatively small contribution of this group to the overall number of infections.

## S3. Back-calculation model

We extended the standard back calculation approach to explicitly model the at-risk population in which new infections arise. We first review the standard model ^7^. For a disease with long incubation period between infection and the development of the disease endpoint, let $t_{0}<t_{1}\ldots<t_{N}$ partition time between $t_{0}$ and $t_{N}$ into intervals of equal length. In discrete time, the convolution

$$\mu_{i}=\sum_{j=0}^{i} h_{j}f_{j-i}$$

links the three components of the back-calculation: $\mu_{i}$, the expected number of occurrences of the end point of interest in the time interval [$t_{i}, t_{i+1}]$;,$h_{j}$ the expected number of new infections during [$t_{j}, t_{j+1}]$; and $f_{j-i},$the probability that infections in the interval [$t_{j}, t_{j+1}]$ experience the end point in [$t_{i}, t_{i+1}]$ for $i=1\ldots N-1$.

It is typically assumed that data on $\mu_{i}$ are available over time; the incubation time distribution $f_{j-i}$, is known from external sources and the $h_{j}$ is unknown and estimated from information on the other two components. If, as usually assumed, infections occur according to a time non-homogeneous Poisson process, the number of new infections in an interval is a Poisson distributed random variable. Making the Poisson assumption in the convolution above, $h_{j}$ represents the expectation of such Poisson variable for interval $[t_{j}, t_{j+1}]$ and, as consequence, the number of endpoints in a later interval [$t_{i}, t_{i+1}]$ is also Poisson distributed with mean $\mu_{i}$ . The expected number of new infections, $h_{j}$, is usually expressed as a smooth function in terms of parameters. Parameters can be estimated by maximising the Poisson likelihood of the data or through a Bayesian approach, by calculating the posterior distribution of the parameters through the combination of the information from the prior distributions and the likelihood.

In the model of Figure 1 in the main text, the distribution $f_{j-i}$ is replaced by the progression through disease states expressed by the progression probabilities and, more importantly, the process of initiating injection now follows a Poisson process with $\gamma_{l}$ being the expected number of new PWID in interval $\left[ t_{l}, t_{l+1} \right]$ and $h_{j}=n_{j}\alpha_{j}$ the expected number of new HCV infection arising in PWID through practicing injecting where $n_{j}$ is the number of susceptible and $\alpha_{j}$ the probability of infection within a single time interval.

### Modelling framework

Modelling is on the basis of 5-year birth cohorts: 1930-1934, 1935-1939 and so on. These groups are tracked over time from birth up to age 89. Each year, the total number of new PWID entering the model is distributed across the birth cohorts according to the age at first use distribution obtained from the UAM (see section A4). The probability of infection in the first year is applied to these new PWID, and they move to infected or susceptible long-term PWID groups accordingly in the next time step. Short-term users that permanently cease injecting within one year move immediately to the infected or susceptible ex-PWID groups (see section A2). Susceptible PWID are then subject to the post-first year risk in subsequent years, and move to the ex-PWID group according to the specified rate of permanent injecting cessation.

Over time, the infected populations within each birth cohort progress through mild, moderate and cirrhosis states according to the annual age-specific probabilities of disease progression, then to ESLD or HCC. The latter are the quantities for which observed data are available, and the total in each birth cohort is related to the birth cohort-specific HES data.

To summarise the model dynamics, the recursive formula for the number of current PWID $c_{t}$ at time *t* is specified as

$c_{t}=c_{t-1}\left( 1-\alpha\right)\left( 1-\kappa\right)+0.66\eta_{t}$,

where $c_{t-1}$ is the number of current PWID in the previous time interval, $\alpha$ is the mortality rate, $\kappa$ the rate of injecting cessation and $\eta_{t}$the number of new injecting initiates, 66% of which remain long-term PWID. The number of ex-PWID $e_{t}$ is specified as

$e_{t}=e_{t-1}\left( 1-\alpha\right)+c_{t-1}\kappa\left( 1-\alpha\right)+0.34\eta_{t}$,

such that ex-PWID remain ex-PWID until they die, current PWID in time *t*-1 that cease injecting move into the PWID group, and 34% of new initiates that cease injecting within one year immediately enter the ex-PWID group.

The number of individuals in disease state *i* at time *t* is defined by

$n_{i,t}=n_{i,t-1}\left( 1-p_{i} \right)\left( 1-\alpha\right)+n_{i-1,t-1}p_{i-1}\left( 1-\alpha\right)$,

where $p_{i}$ is the probability of progressing from disease state *i* to *i*+1 (which includes susceptible to infected states), $n_{i-1,t-1}$ the number of individuals at time *t*-1 in the preceding disease state, *i*-1, and $p_{i-1}$the probability of progressing from the previous disease state to state *i*.

For simplicity, these equations do not include indexing of the age-specific parameters for disease progression and mortality, and differential mortality in different risk groups. Those that die are removed from the model, as are those reaching ESLD or HCC states, which are considered to be terminating states.

In the model, these transition dynamics are combined to include progression through disease states and movement from current to ex-PWID groups, which are assumed to be independent processes and therefore involve multiplications of the transition probabilities specified above. Similarly, the processes of diagnosis and treatment occur independently of disease stage and risk group, in the absence of any information on which groups are diagnosed/treated. These assumptions could be relaxed to include risk-group specific probabilities of disease progression, diagnosis and treatment or other complexities as required, if information were available.

## 4. Model parameters

### Initiation of injecting drug use

The underlying Poisson process for recruitment of new PWID is modelled as a piecewise constant function with 5-years intervals from 1940-1970, 2-year intervals from 1970-1980 and 1-year intervals thereafter. The prior distribution for each rate is assigned an uninformative normal distribution on the log scale, and some smoothing imposed on the rates over time via a 2^nd^ order random walk, which penalises local non-linearity:

$\beta_{t}\sim N(2\beta_{t-1}-\beta_{t-2},\sigma^{2})$,

where $\beta_{t}=log(\gamma_{t})$ is the log of the total number of new PWID in year *t* and $\sigma^{2}$ chosen to provide a “reasonable” degree of smoothing. With *σ* =0.1, 95% of the probability mass for the prior of $\beta_{t}$ lies within a ratio of 0.82 to 1.22 of the linear trend over time from *t*-2 to *t*-1.

### Progression probabilities

We use information on pre-cirrhotic disease progression from the Trent HCV cohort, a study of HCV-infected patients referred to hospitals in the Trent region from 1991-2003 ^8^. Information from the study consisted of estimated annual probabilities and confidence intervals for progression from mild to moderate and moderate to compensated cirrhosis in HCV-infected individuals aged 0-29, 30-39, 40-49 and 50+. These estimates are used as informative priors and assigned a normal distribution on the logit scale reflecting the degree of uncertainty in the published estimates. Ages 0-29 are assumed to have the same rate of progression, as there would be little information to determine otherwise; ages 50-59, 60-69 and 70-89 are assigned separate priors based on the 50+ estimate.

Estimates of disease progression from cirrhosis to ESLD and HCC are based on estimates from other studies of disease progression and previous modelling ^3,9–11^. Priors for ESLD were the same across age groups. Progression to HCC was assumed to follow a log-linear change with age, as in previous modelling ^3,12^.

### Injecting cessation

Sweeting et al ^13^ estimated the average duration of injecting before permanent cessation, with a mean of 11 years and 95% credible interval of 6.9-19.6. This included 34% of those initiating injecting ceasing within one year, and implies that the remaining long-term PWID have a mean duration of around 16 years. We fixed the first year cessation rate at 34% and specified a beta(2,4) distribution for long-term injecting duration over the range 7 to 21 years, which approximately reflects the distribution above. The resulting average injecting duration *D* is converted to an annual cessation rate in the model, i.e., *κ*=1/*D*.

Allowing flexibility in both disease progression probabilities and injecting duration results in undesirable feedback between the disease progression process and injecting duration: the latter has very weak information and can be altered substantially to provide a better fit to the endpoint data. One alternative is to assign a stronger prior, or even a fixed value, for injecting duration; however, this does not account for the uncertainty in this quantity. We therefore restrict the flow of information in the model such that the injecting duration parameter includes the specified level of uncertainty, but is not altered by the data.

### Infection in PWID

The baseline force of infection in PWID and hazard ratio for 1^st^ year vs. subsequent infection risk are assigned non-informative normal priors on the log scale (see section A2 for further details).

### Known quantities

The age distribution of new injectors is based on UAM data ^14^.

We use ONS mortality rates for non-HCV mortality in the never-injecting risk groups ^15^, but up to 5 times higher in younger age groups in those ever injecting drugs due to additional comorbidities in these group ^12^. While currently injecting, we assume an additional risk due drug overdose and use age-specific rates comparable with those observed in the Edinburgh Addiction Cohort ^16^.

SVR rates under interferon-based therapies are based on published estimates ^17^, new therapies are assumed to have a 90% intention-to-treat success rate in all individuals ^18^. Those achieving SVR in mild and moderate states were assumed to have no further disease progression (unless re-infected), and those with cirrhosis reduced disease progression by a fixed risk ratio of 0.08 for ESLD and 0.27 for HCC ^19^.

### Background infection rate in non-PWID

Unlike the UAM survey of PWID, there are no seroprevalence data specifically for those that have never injected drugs in the general population. Survey data will tend to include a mixture of those who have ever and never injected drugs, and this mixture is generally unknown or difficult to quantify. Careful modelling of the mixture of these populations and the use of additional data sources is required to obtain an unbiased estimate, such as multi-parameter evidence synthesis (MPES) ^20^. We therefore used age-specific MPES estimates of HCV prevalence in those never injecting drugs of white/other and South Asian ethnicity ^21^. We assumed a uniform distribution of age within each age group, and specified a force of infection model to relate age-specific antibody prevalence to the annual rate of chronic infection, with the form

$S\left( a \right)={exp(-\lambda a)}/{0.76}$,

where *S*(*a*) is the proportion without antibodies at age *a*, *λ* is a constant rate of chronic infection and 0.76 the expected proportion of chronic infection in those with antibodies ^22^. Although an age-specific rate (across broad groups) could be obtained from the age-specific prevalence estimates, there was no evidence that the rate varied with age. We further assumed a constant rate over time, in the absence of information at different time points ^6^, but assumed that the rate was reduced by 40% in those of white/other ethnicity from 1991, following the introduction of blood donation screening ^23^. The reduction was assumed to be 20% in South Asians post-1991, as a greater proportion of infections arise outside the UK ^24^. These are somewhat arbitrary assumptions, although the never-injecting risk groups are a relatively small part of the epidemic.

### Numbers diagnosed and treated

Rates of diagnosis were not explicitly modelled: for each iteration of the MCMC simulation numbers of observed new diagnoses were divided by the total number of undiagnosed individuals to obtain the probability of diagnosis for each year/age group, constrained to a maximum of 0.8 of the remaining pool, and the resulting proportion move from undiagnosed to undiagnosed states accordingly. A similar approach was applied to the number of treatment initiations arising from the pool of diagnosed individuals. Figure S3 shows how individuals move through the state of susceptible, infected but never diagnosed, diagnosed and achieving SVR. Those in the SVR state may become re-infected according and return to the infected, ever-diagnosed state.

Figure S3. Movement through susceptible, infected, diagnosed and treated states in the model.

A summary of the model parameters is given in Table S5.

**Table S5**. Parameters in the model, which are a mixture of fixed values and estimated quantities. The latter are assigned prior distributions and shown below in bold.

| Parameter | Range/group | Value/prior | Description |
| --- | --- | --- | --- |
| *PWID population parameters* | | | |
| **Rate of new PWID** | All years | β_t_ ~ N(2β_t-1_-β_t-2_, 0.1) | 2nd order random walk on log scale. |
| Age distribution at injecting initiation | Age 13-16 | 18.70% | UAM data on PWID, 2000-2014. Reported age at first use. |
|  | Age 17-20 | 35.20% |  |
|  | Age 21-24 | 20.70% |  |
|  | Age 25-29 | 15.50% |  |
|  | Age 30-34 | 6.00% |  |
|  | Age 35-39 | 2.60% |  |
|  | Age 40-49 | 1.30% |  |
| Cessation within one year of starting injecting | All ages | 34% | Based on Sweeting 2009. |
| **Permanent cessation in long-term PWID** | All ages | 1 / (Beta(2,4)*15+7) | Based on Sweeting 2009; results in mean injecting durations of 7-21 years, with greater probability around 12 years. |
| *Annual probabilities of infection* | | | |
| **Force of infection in PWID** | Injecting > 1 year | N(-3.5,5) | Uninformative priors on the log scale for the baselined FOI in each 5-year time period. |
|  | 1st year HR | N(0,5) | Uninformative priors on the log scale for the 1st year hazard ratio. |
| Background rate of infection (non-injecting drug use) | White/other, pre-1991 | 0.00001 | Constant force of infection based on age-specific prevalence estimates from MPES (Harris 2011) and impact of blood screening from 1991 (Deuffic et al, 2004) |
|  | White/other, post-1991 | 0.000006 |  |
|  | South Asians, pre-1991 | 0.00015 |  |
|  | South Asians, post-1991 | 0.00012 |  |
| *Disease progression probabilities* | | | |
| **Mild to moderate chronic** | Age 0-29 | 0.012 (0.010-0.016) | Annual progression probabilities based on the Trent Cohort (Sweeting et al 2006). Priors for age groups over 50 are the same, but allowed to vary in the model. |
|  | Age 30-39 | 0.027 (0.017-0.041) |  |
|  | Age 40-49 | 0.040 (0.021-0.071) |  |
|  | Age 50-59 | 0.128 (0.075-0.200) |  |
|  | Age 60-69 | 0.128 (0.075-0.200) |  |
|  | Age 70+ | 0.128 (0.075-0.200) |  |
| **Moderate chronic to compensated cirrhosis** | Age 0-29 | 0.029 (0.018-0.045) | Annual progression probabilities based on the Trent Cohort (Sweeting et al 2006). Priors for age groups over 50 are the same, but allowed to vary in the model. |
|  | Age 30-39 | 0.075 (0.028-0.191) |  |
|  | Age 40-49 | 0.048 (0.015-0.145) |  |
|  | Age 50-59 | 0.073 (0.023-0.218) |  |
|  | Age 60-69 | 0.073 (0.023-0.218) |  |
|  | Age 70+ | 0.073 (0.023-0.218) |  |
| **Compensated cirrhosis to ESLD** | All ages | 0.065 (0.040-0.095) | Pooled estimate from Hutchinson et al 2005, used as prior in previous modelling. Same priors for each age group but allowed to vary by age. |
| **Compensated cirrhosis to HCC** | Age 0-29 | 0.008 (0.004-0.016) | Age-specific estimate used as prior in previous modelling work (Harris 2014), log-linear increase with age. |
|  | Age 30-39 | 0.013 (0.008-0.022) |  |
|  | Age 40-49 | 0.021 (0.014-0.031) |  |
|  | Age 50-59 | 0.035 (0.025-0.048) |  |
|  | Age 60-69 | 0.057 (0.038-0.079) |  |
|  | Age 70-89 | 0.091 (0.056-0.147) |  |
| RR for post-SVR progression | ESLD | 0.08 | Risk ratio for disease progression in those achieving SVR while in compensated cirrhosis stage |
|  | HCC | 0.27 |  |
| *Diagnosis and treatment parameters* | | | |
| **Diagnosis** | Age and time-specific | As observed | Rate calculated based on observed diagnosis and estimated undiagnosed prevalence. |
| **Treatment** | Time-specific | As observed | Rate calculated based on observed treatment and estimated diagnosed prevalence. |
| SVR rates for IFN-based treatment | Mild | 0.55-0.83 | Fixed age-specific rates from Thompson et al; SVR rates decrease with age, given here are the lower and upper estimates. |
|  | Moderate | 0.41-0.69 |  |
|  | Cirrhosis | 0.19-0.37 |  |
| SVR rates for new DAAs | Mild | 0.9 | Harris et al 2014. Constant across age groups. |
|  | Moderate | 0.9 |  |
|  | Cirrhosis | 0.9 |  |
| *Annual mortality rates* | | | |
| Ever-PWID non-HCV mortality | Age 0-29 | 0.001 | ONS mortality rates increased by a factor of 2, and up to 5 in younger age groups, as in previous modelling, reflecting the higher mortality in ever-PWID due to other co-morbidities. |
|  | Age 30-49 | 0.003 |  |
|  | Age 50-59 | 0.009 |  |
|  | Age 60-69 | 0.023 |  |
|  | Age 70-79 | 0.057 |  |
|  | Age 80-89 | 0.12 |  |
| Excess mortality while currently injecting | All ages | 0.005-0.0350 | Additional mortality risk due to drug-related death, increasing with age (13) |
| Never-PWID non-HCV mortality | Age 0-29 | 0.0002 | ONS mortality rates as in the general population. |
|  | Age 30-49 | 0.0014 |  |
|  | Age 50-59 | 0.0048 |  |
|  | Age 60-69 | 0.0117 |  |
|  | Age 70-79 | 0.0283 |  |
|  | Age 80-89 | 0.0601 |  |

## 5. Estimation

The data sources used in the model are counts of disease endpoint data, data on HCV prevalence in PWID and data on the proportion of PWID in the population. Each data source has an independent Poisson or binomial likelihood, and the contributions of the kernel log-likelihoods are summed to produce the overall likelihood.

The log-likelihood contributions are as follows:

1) HES data on ESLD and HCC by calendar year and age. The disease endpoint data $y_{i}$for each year and birth cohort combination *i* follow a non-homogeneous Poisson process with mean $\mu_{i}$, the latter being defined by a function and disease progression probabilities that link initiation of injecting drug use and infection to the development of ESLD and HCC. The kernel log-likelihood for the HES data are defined as

${LL}_{\text{HES}}=\sum_{i=1}^{n} y_{i}\log\left( \mu_{i} \right)-\mu_{i}$,

where *i* is for all birth cohort/time combinations and ESLD and HCC outcomes available from HES data.

2) Data on the number of PWID are expressed as binomial data representing proportions of the adult England population. This approach is similar to previous back-calculation modelling, which incorporated estimates of chronic HCV prevalence in the likelihood as binomial data ^3,12^.

The proportion of the population aged 15-64 that are current PWID is a function of the parameters for the number initiating injecting drug use over time, the cessation rate and mortality in this population, which includes HCV-related mortality due to disease progression. Defining this proportion in 2011 as $p$, and with *r* and *n* the numerator and denominator that provide a population-level proportion with appropriate uncertainty for the external information on PWID prevalence, the resulting binomial kernel log-likelihood is

${LL}_{\text{Pop}}=\log\left( p \right)r+\log\left( 1-p \right)(n-r)$.

3) UAM prevalence data according to calendar year and injecting duration. Estimates of the rate of infection in the 1^st^ year and subsequent years of injecting *a*, varying by calendar time *t*, are related to HCV prevalence according to the force of infection model as defined in section A2.With $r_{i}$ the observed number of infections and $n_{i}$ the number of individuals for injecting duration/time combination *i* in the UAM data, and *p_i_* the proportion infected from the model, the binomial kernel log-likelihood is

${LL}_{\text{UAM}}=\sum_{i=1}^{n} \log\left( p_{i} \right)r_{i}+\log\left( 1-p_{i} \right)(n_{i}-r_{i})$.

The likelihood contributions from (1), (2) and (3) are summed to give the total log-likelihood *LL*.

The diagnosis and treatment data are used in the model, but do not inform the likelihood. The total number of individuals in each age group moving from undiagnosed to diagnosed states is simply the observed number of new diagnoses, up to a maximum of 80% of the undiagnosed pool, although in practice the limit is never reached in the model and there are always sufficient undiagnosed infections to produce the observed number of new diagnoses. A similar approach is used for the treatment data, with new treatment initiations arising from the diagnosed pool.

Model fitting was conducted in Stata version 13 (Stata Corp., College Station, TX). Initial attempts to fit models in the Bayesian model-fitting software WinBUGS were problematic, due to the highly recursive nature of back-calculation and the large number of states and age-time combinations. Model updating and likelihood calculation procedures were optimised to make use of Stata’s efficient data manipulation routines. By aggregating birth cohorts to 5-year intervals rather than individual years, the number of computations is reduced by 80%.

We used a simple component-wise Metropolis-Hastings sampling algorithm to obtain posterior distributions of parameters and functions thereof. At each iteration of the simulation process, the parameters are sampled in turn. Based on the current value of a parameter $\theta$, a proposed new value is generated $\theta_{\text{cand}}=\theta+\delta$, where $\delta$ is uniformly distributed over an interval [-*a*, *a*] (see below). The probability of accepting the proposed value for the parameter is defined as

$\text{pr}\left( accept \right)=\min[1, exp(LL(\theta_{cand})-LL(\theta))p(\theta_{cand})/p(\theta)]$,

where $p(\theta)$and $p(\theta_{cand})$ are the density of the prior distribution for the parameter at the current and proposed values respectively. Values of *a*, which determines the step size for proposals, are set to produce acceptance probabilities of approximately 30% for each parameter once the model has converged.

## References

1. People who inject drugs: HIV and viral hepatitis monitoring. 2017. https://www.gov.uk/government/statistics/people-who-inject-drugs-hiv-and-viral-hepatitis-monitoring.

2. Laboratory reports of hepatitis A and C: 2017. 2018. https://www.gov.uk/government/publications/laboratory-reports-of-hepatitis-a-and-c-2017.

3. Harris RJ, Thomas B, Griffiths J, et al. Increased uptake and new therapies are needed to avert rising hepatitis C-related end stage liver disease in England: modelling the predicted impact of treatment under different scenarios. *J Hepatol*. 2014;61:530-537.

4. Lattimore S, Irving W, Collins S, et al. Using surveillance data to determine treatment rates and outcomes for patients with chronic hepatitis C virus infection. *Hepatology*. 2014;59(4):1343-1350. doi:10.1002/hep.26926.

5. Farrington CP, Kanaan MN, Gay NJ. Estimation of the basic reproduction number for infectious diseases from age-stratitified serological survey data. *J Appl Stat*. 2001;50(3):251-292.

6. Ades AE, Nokes DJ. Modeling Age- and Time-Specific Incidence from Seroprevalence: Toxoplasmosis. *Am J Epidemiol*. 1993;137(9):1022-1034. http://www.ncbi.nlm.nih.gov/pubmed/8317447.

7. Brookmeyer R, Gail MH. *AIDS Epidemiology: A Quantitative Approach*. Oxford University Press; 1994.

8. Sweeting MJ, De Angelis D, Neal KR, et al. Estimated progression rates in three United Kingdom hepatitis C cohorts differed according to method of recruitment. *J Clin Epidemiol*. 2006;59(2):144-152. doi:10.1016/j.jclinepi.2005.06.008.

9. Hutchinson SJ, Bird SM, Goldberg DJ. Modeling the current and future disease burden of hepatitis C among injection drug users in Scotland. *Hepatology*. 2005;42(3):711-723. doi:10.1002/hep.20836.

10. Fattovich G, Giustina G, Degos F, et al. Morbidity and mortality in compensated cirrhosis type C: A retrospective follow-up study of 384 patients. *Gastroenterology*. 1997;112(2):463-472. doi:10.1053/gast.1997.v112.pm9024300.

11. Fattovich G, Stroffolini T, Zagni I, Donato F. Hepatocellular carcinoma in cirrhosis: incidence and risk factors. *Gastroenterology*. 2004;127(5 Suppl 1):S35-S50. http://www.ncbi.nlm.nih.gov/pubmed/15508101.

12. Sweeting MJ, De Angelis D, Brant LJ, Harris HE, Mann a G, Ramsay ME. The burden of hepatitis C in England. *J Viral Hepat*. 2007;14(8):570-576. doi:10.1111/j.1365-2893.2007.00851.x.

13. Sweeting MJ, De Angelis D, Ades A, Hickman M. Estimating the prevalence of ex-injecting drug use in the population. *Stat Methods Med Res*. 2009;18(4):381-395. doi:10.1177/0962280208094704.

14. Kessel AS, Watts CJ. Evaluation of the unlinked anonymous prevalence monitoring programme for HIV in England and Wales: science, ethics and health policy. *Med Sci Monit Int Med J Exp Clin Res*. 2001;7(5):1052-1063. http://www.ncbi.nlm.nih.gov/pubmed/11535957.

15. Office of National Statistics. *B2.1 2012-Based Mortality Rates (qx), 1981-2062, Principal Projection, Great Britain, Revised 02-Apr-2014*.; 2014. http://ons.gov.uk/ons/taxonomy/index.html?nscl=Life+Expectancies#tab-data-tables.

16. Kimber J, Copeland L, Hickman M, et al. Survival and cessation in injecting drug users: Prospective observational study of outcomes and effect of opiate substitution treatment. *BMJ*. 2010;341(7764):135. doi:10.1136/bmj.c3172.

17. Thomson BJ, Kwong G, Ratib S, et al. Response rates to combination therapy for chronic HCV infection in a clinical setting and derivation of probability tables for individual patient management. *J Viral Hepat*. 2008;15(4):271-278. doi:10.1111/j.1365-2893.2007.00941.x.

18. Kowdley K V., Gordon SC, Reddy KR, et al. Ledipasvir and Sofosbuvir for 8 or 12 Weeks for Chronic HCV without Cirrhosis. *N Engl J Med*. 2014;370(20):1879-1888. doi:10.1056/NEJMoa1402355.

19. Singal AG, Volk ML, Jensen D, Di Bisceglie AM, Schoenfeld PS. A sustained viral response is associated with reduced liver-related morbidity and mortality in patients with hepatitis C virus. *Clin Gastroenterol Hepatol*. 2010;8(3):280-288.e1. doi:10.1016/j.cgh.2009.11.018.

20. Sweeting M, De Angelis D, Hickman M, Ades a E. Estimating hepatitis C prevalence in England and Wales by synthesizing evidence from multiple data sources. Assessing data conflict and model fit. *Biostatistics*. 2008;9(4):715-734. doi:10.1093/biostatistics/kxn004.

21. Harris RJ, Ramsay M, Hope VD, et al. Hepatitis C prevalence in England remains low and varies by ethnicity: an updated evidence synthesis. *Eur J Public Health*. 2012;22(2):187-192. doi:10.1093/eurpub/ckr083.

22. Micallef JM, Kaldor JM, Dore GJ. Spontaneous viral clearance following acute hepatitis C infection: a systematic review of longitudinal studies. *J Viral Hepat*. 2006;13(1):34-41. doi:10.1111/j.1365-2893.2005.00651.x.

23. Deuffic-Burban S, Wong JB, Valleron AJ, Costagliola D, Delfraissy JF, Poynard T. Comparing the public health burden of chronic hepatitis C and HIV infection in France. *J Hepatol*. 2004;40(2):319-326. doi:10.1016/j.jhep.2003.10.018.

24. Uddin G, Shoeb D, Solaiman S, et al. Prevalence of chronic viral hepatitis in people of south Asian ethnicity living in England: the prevalence cannot necessarily be predicted from the prevalence in the country of origin. *J Viral Hepat*. 2010;17(5):327-335. doi:10.1111/j.1365-2893.2009.01240.x.
